# Experiences of Persons with Mild Dementia and Their Caregivers in a Caregiver‐Assisted Combined Exercise and Cognitive Intervention: A Qualitative Study

**DOI:** 10.1002/alz70858_098126

**Published:** 2025-12-24

**Authors:** XUE Dandan, Ling Yue, Yanmei Wang, Meiqing Sheng

**Affiliations:** ^1^ Department of Nursing, Gongli Hospital of Shanghai Pudong New Area, Shanghai, China; ^2^ Shanghai Alzheimer's Disease and Related Disorders Center, Shanghai Jiao Tong University, Shanghai, China; ^3^ Shanghai Mental Health Center, Shanghai Jiao Tong University School of Medicine, Shanghai, China

## Abstract

**Background:**

A caregiver‐assisted combined exercise and cognitive intervention was developed and pilot tested for its effect on health outcomes in persons with mild dementia (PwMD). A qualitative study was conducted to explore the acceptability, engagement experience and perceived benefits from the perspectives of PwMD and their caregivers.

**Methods:**

This study involved older adults with confirmed mild dementia who were able to engage in physical activity, literate, and had a family caregiver willing to participate. Individual semi‐structured qualitative interviews were conducted with the seven dyads who engaged in the tested intervention. An interview guide with open‐ended questions and probes were used to facilitate the interviews. All the interviews were audio‐taped, transcribed verbatim, and analyzed using content analysis.

**Results:**

Five themes were emerged: (1) shift from hesitation or passivity to proactive engagement, (2) high acceptability of the intervention design and materials, (3) improved cognitive, psychological symptoms and activity levels of PwMD, (4) improved knowledge, attitudes and dyadic relationships among caregivers, and (5) beliefs motivate while task difficulties and caregiver constraints act as barriers. PwMD's attitudes toward the intervention shifted from initial reluctance to active engagement, while caregivers changed from passive receivers to active agents. The dyads expressed satisfaction with the intervention design and indicated that the intervention materials could help guide, monitor and facilitate self‐practice. However, some dyads recommended tailoring the training to fit the educational background and levels of impairment in each cognitive domain of the PwMD. They also suggested enhancing the diversity and accessibility of the intervention. PwMD perceived the intervention was effective to improve their cognitive function, neuropsychiatric symptoms, and physical and cognitive activity levels. Caregivers reported increased knowledge and skills, improved mood, more positive attitudes towards the disease, and strengthened dyadic relationships. The dyads’ belief in the benefits of the intervention facilitated the exercise and cognitive training of PwMD. However, PwMD faced difficulties in completing tasks like repeating short texts, and caregivers sometimes struggled with patience and time constraints when assisting participants in training.

**Conclusions:**

The findings implied the combined intervention was feasible and beneficial for the dyads. A full‐scale study is warranted to further test its effects.

